# F236. CLONIDINE NORMALIZES MMN IN SCHIZOPHRENIA PATIENTS ON STABLE MEDICATION

**DOI:** 10.1093/schbul/sby017.767

**Published:** 2018-04-01

**Authors:** Caitlyn Kruiper, Birte Glenthoj, Bob Oranje

**Affiliations:** 1 Center for Neuropsychiatric Schizophrenia Research (CNSR) and Center for Clinical Intervention and Neuropsychiatric Schizophrenia Research (CINS), Mental Health Center Glostrup, University of Copenhagen, University Medical Center Utrecht, Utrecht University; 2 Center for Neuropsychiatric Schizophrenia Research (CNSR) Center for Clinical Intervention and Neuropsychiatric Schizophrenia Research (CINS), Copenhagen University Hospital, Psychiatric Center Glostrup

## Abstract

**Background:**

Schizophrenia is a severe brain disorder with profound deficits in prefrontal cortical cognitive functioning. These cognitive deficits form a core feature in schizophrenia for which treatment has been proven to be clinically challenging. One of the key neurotransmitters involved in cognitive functioning is noradrenaline. Previous research has demonstrated disrupted noradrenergic activity in schizophrenia while several studies report improvements in prefrontal cognitive functioning by a selective α2-agonist. Clonidine is such a selective α2A-agonist and previous research in our lab has demonstrated that a (range of) single dosage(s) of clonidine normalize(s) sensory gating in chronically ill, yet stably medicated patients with schizophrenia. Currently, we investigated if clonidine also normalizes Mismatch Negativity (MMN) deficits in this same group of patients. This is important, since reports have shown that MMN amplitude is associated with negative symptoms and cognitive functioning in schizophrenia.

**Methods:**

In a pseudo-randomized, double-blind, placebo-controlled experiment twenty chronically ill, yet stably medicated, male patients with schizophrenia were tested with the MMN paradigm from the Copenhagen Psychophysiological Test-Battery (CPTB) on 5 different occasions, each separated by a week. Four hours prior to testing patients were randomized administered either a placebo (non psycho-active compound) or a single dose of 25, 50, 75 or 150 μg of clonidine (Catapresan) on top of their usual medication on each occasion, in such a way that each patient received every dose once. Patients were matched on age and gender with 20 healthy controls (HC), who did not receive any treatment. The MMN paradigm consisted of 1800 stimuli with 4 types of stimuli: 1 standard (1000 Hz, 50 ms) presented 82% of the time and 3 types of deviants, based on either frequency (FreqMMN: 1200 Hz, 50 ms), duration (DurMMN: 1000 Hz, 100 ms) or their combination (FreqDurMMN: 1200 Hz, 100 ms) each with a probability of 6%. All stimuli had an intensity of 75dB and were presented with an interstimulus interval randomized between 300 and 500 ms. Subjects were requested to ignore all stimuli and were therefore watching a (muted) nature documentary.

**Results:**

In the placebo condition, patients had significantly reduced DurMMN and FreqDurMMN amplitude compared to HC, whereas FreqMMN did not show significant group differences. Furthermore, all doses of clonidine normalized FreqDurMMN amplitude in such a way that it was not significantly different anymore from the HC, while DurMMN only normalized with the highest dose (150ug).

**Discussion:**

Our results indicate disrupted MMN amplitude in chronically ill patients with schizophrenia in spite of the fact that they were stable on their medical treatment. In addition, our data provide evidence that a single dose of clonidine is able to normalize MMN amplitude in these patients. Furthermore, patients could not distinguish between the placebo and the treatment conditions or reported any side effects of these low doses of clonidine. Together with our previous reports indicating normalized sensory and sensorimotor gating in these patients following administration of clonidine, our results could be of potential high clinical relevance in the treatment of schizophrenia. Future studies should therefore focus on longer trial periods to investigate if clonidine, besides normalizing MMN amplitude and sensory(motor) gating, can also ameliorate negative symptoms and cognitive functioning in schizophrenia.

